# Simple sequence repeat analysis of new potato varieties developed in Alberta, Canada

**DOI:** 10.1002/pld3.140

**Published:** 2019-06-05

**Authors:** Anne‐Sophie Tillault, Dmytro P. Yevtushenko

**Affiliations:** ^1^ Department of Biological Sciences University of Lethbridge Lethbridge Alberta Canada

**Keywords:** DNA fingerprinting, polymorphism, potato varieties identification, single sequence repeat, *Solanum tuberosum* L., SSR markers

## Abstract

The worldwide demand for potato production requires the constant development of new potato varieties with improved yield, quality, disease resistance, and abiotic tolerance. However, cultivar registration is preceded by a long process to morphologically and physiologically characterize the plants. Notably, this process can be expedited by DNA marker analysis. Simple sequence repeats (SSRs), also known as microsatellites, are the most common reliable DNA markers used to discriminate between genotypes. In this study, 20 potato varieties, including five new genotypes developed in Alberta, Canada, were fingerprinted using 10 SSR markers selected for their high discriminatory power. Different SSRs were amplified from potato DNA using specific primers, and the DNA fragment sizes were analyzed by denaturing polyacrylamide gel electrophoresis. The number of alleles per locus ranged from two for the SSR marker STPoAc58 to six for STM0030 and STM0037 with an average of 4.4. In addition, a total of 77 unique patterns were observed for the 10 SSR markers. The polymorphic information content ranged from 0.477 to 0.802 with an average of 0.675 per locus. In this study, STM0037, STM1016, and STM1104 were found to be the best SSR markers to detect genetic differences between potato varieties. A minimum of two markers was required to distinguish between all 20 genotypes. Most importantly, this highly informative molecular tool confirmed that the developed potato varieties were genetically different from their respective maternal lines and potentially constituted new cultivars.

## 1 INTRODUCTION

The cultivated potato (*Solanum tuberosum* L.) is the most important non‐cereal crop in the world and a key component of global food security (Devaux, Kromann, & Ortiz, 2014). More than 4,000 different potato varieties are grown worldwide (Zaheer & Akhtar, 2016). The increase in global potato production requires the constant development of new varieties to satisfy the demands of the consumer (e.g., improved taste and nutritional value), the grower (e.g., higher yield and enhanced disease resistance), and the processor (e.g., low reducing sugars) (Govindaraj, Vetriventhan, & Srinivasan, 2015). Traditionally, potato cultivars are differentiated and assessed using morphological and physiological characteristics, such as plant architecture, flower color, disease resistance, and sprout and tuber types (van Eck, 2007). However, these traits can be affected by the environment, resulting in potential misidentification (Govindaraj et al., 2015). In addition, traditional methods of cultivar classification are largely subjective, time consuming, and require an extensive set of skills.

The correct identification of potato varieties is an important and necessary component of breeding programs, seed certification, germplasm management, new cultivar registration, intellectual property rights, and trademark. Thus, it is critical to develop a rapid and reliable method to assess the genetic diversity among potato varieties.

DNA markers, also called molecular markers, are currently the most widely used genetic markers for potato variety identification due to their distinct advantages over morphological and biochemical markers (Gebhardt, 2007; Govindaraj et al., 2015). Indeed, they are independent from the environment, usually phenotypically neutral, show Mendelian inheritance, and are present in any tissue irrespective of the plant growth stage (Govindaraj et al., 2015). Different types of DNA markers have been extensively studied over the past three decades, including random amplified polymorphic DNA, restriction fragment length polymorphism, amplified fragment length polymorphism, single nucleotide polymorphism, inter‐simple sequence repeat, and simple sequence repeat (SSR) (McGregor, Lambert, Greyling, Louw, & Warnich, 2000). Although each type of DNA marker has its own advantages, SSRs, also called microsatellites, have been demonstrated to be the best choice for fingerprinting and evaluating genetic diversity in plants at the species level (Milbourne et al., 1998; Powell et al., 1996; Russell et al., 1997). Simple sequence repeats are repetitions of short tandem DNA motifs (1–6 nucleotides) generated by DNA polymerase slippage, mismatch/double strand break repair, unequal crossing‐over, and retrotransposition (Vieira, Santini, Diniz, & de Munhoz, 2016). The SSRs have desirable characteristics as DNA markers because they (a) are abundant, (b) are codominant markers, (c) demonstrate reproducibility, (d) show a high level of polymorphism, (e) possess a specific chromosome location (if mapped), (f) extensively cover the genome (including organelle DNA), and (g) are easily analyzed with polymerase chain reaction (PCR) (Kalia, Rai, Kalia, Singh, & Dhawan, 2011). Moreover, the sequences flanking an SSR and used to design specific primers are generally conserved in closely related species (Kalia et al., 2011). These characteristics make SSRs a powerful tool for genetic applications, such as genome mapping, diversity analysis, phylogenetic studies of some species, cultivar discrimination, as well as marker‐assisted selection in breeding programs (Vieira et al., 2016).

In potatoes, several studies have been conducted to identify and select appropriate SSRs by detecting specific repeat motifs or using enriched genomic libraries and a database of expressed sequence tags (Ashkenazi et al., 2001; Feingold, Lloyd, Norero, Bonierbale, & Lorenzen, 2005; Ghislain et al., 2004, 2009; Milbourne et al., 1998; Provan, Powell, & Waugh, 1996; Veilleux, Shen, & Paz, 1995). Nowadays, whole genome sequencing is used for this purpose. A potato genetic identification (PGI) kit that contains the best 24 SSR markers of 148 SSRs tested with 742 landraces has been developed (Ghislain et al., 2004, 2009). Following the demonstration that SSR analysis can be used to identify potato cultivars (Kawchuk et al., 1996), SSR markers have been used to assess potato diversity, facilitate breeding programs, and identify germplasm in France (Moisan‐Thiery et al., 2005), Argentina (Ispizúa, Guma, Feingold, & Clausen, 2007), Latvia (Voronova, Veinberga, Skrabule, & Rungis, 2008), Canada (Fu, Peterson, Richards, Tarn, & Percy, 2009), Brazil (Rocha, Paiva, de Carvalho, & Guimarães, 2010), India (Sharma & Nandineni, 2014), China (Liao & Guo, 2014), Iran (Talebi, Salimi, Bahar, & Mirlohi, 2016), and the USA (Bali et al., 2017).

In this study, 10 SSR markers were used to genotype new potato varieties developed by a private breeder in Alberta through the repeated selection of tubers derived from true botanical seeds, as well as their maternal lines. The SSR markers were carefully selected from previous studies based on their polymorphism and discrimination capability. Genotyping was conducted using PCR amplification of genomic DNA with specific primers, followed by the separation of the amplified fragments in denaturing polyacrylamide gel. The goals of this study were (a) to evaluate the power of discrimination of the different SSR markers for future potato genotype analysis and (b) to confirm that the new varieties developed are genetically different from the maternal lines, which is required for cultivar registration and proprietary rights by the breeder.

## 2 MATERIALS AND METHODS

### 2.1 Plant material

Twenty genotypes of cultivated potato (*Solanum tuberosum* L.) were tested in this study, including five potential new varieties generated through open pollination by a private breeder, John Safroniuk (Wetaskiwin), three maternal lines that served as a source of true botanical seeds, and 12 varieties routinely grown by the breeder that may be paternal lines (Table 1). Tubers of the new potato varieties were obtained directly from the private breeder. Two varieties were obtained from Haenni Farms (Millet) and Eagle Creek Seed Potatoes (Bowden). In vitro plants for the remaining potato cultivars were obtained from the Crop Diversification Center North (Alberta Agriculture and Forestry). The chemicals were received from Fisher Scientific (Hampton) and the oligonucleotides from Integrated DNA Technology (IDT).

**Table 1:** List of potato genotypes used in this study including their pedigree, year of selection or release, type, and country of origin

Group	Variety name	Pedigree	Year	Type	Country of origin
1	CW2011	Russet Norkotah x ‐	2011	White	Canada
	N1‐WF	‐ x ‐	2008	Red‐skin	Canada
	OB3	Norland x ‐	2008	Red‐skin	Canada
	OB3‐A	OB3 x ‐	2016	Red‐skin	Canada
	Rose Anna	Banana x ‐	2006	Fingerling	Canada
2	Banana	‐ x ‐	–	Fingerling	Russia
	Norland	Redkote × ND626	1957	Red‐skin	USA
	Russet Norkotah	ND9526‐4 Russ × ND9687‐5 Russ	1987	Russet	USA
3	Alta Blush	‐ x ‐	2013	White	Canada
	Bintje	Munstersen × Fransen	1904	Yellow	Netherlands
	Cecile	Nicola × RZ‐88‐204	1992	Red‐skin	Netherlands
	Cherry Red	ND4750‐2R × LA1858	1999	Red‐skin	USA
	Kennebec	B127 × USDA96‐56	1948	White	USA
	Purple Viking	‐ x ‐	–	Purple	–
	Ranger Russet	Butte × A6595‐3	1991	Russet	USA
	Russet Burbank	Mutant of Burbank	1902	Russet	USA
	Russian Blue	‐ x ‐	–	Purple	Russia
	Shepody	BakeKing × F58050	1980	White	Canada
	Spunta	Bea × USDA 96‐56	1967	Yellow	Netherlands
	Yukon Gold	Norgleam × W 5279‐4	1966	Yellow	Canada

Note

The potato varieties are divided in three groups. Group 1: potential new potato varieties generated from true botanical seeds obtained by open pollination. Group 2: maternal lines used for the breeding. Group 3: potato cultivars grown in breeder's field during the breeding process.

### 2.2 Genomic DNA isolation

A single best‐looking tuber was selected for DNA isolation for each new variety. The tubers were stored at room temperature in the dark for several weeks to initiate sprouting and new leaf growth. Approximately 100 mg of fresh young leaves were collected from sprouting tubers or from in vitro plants, homogenized in liquid nitrogen, and used for genomic DNA extraction with an E.Z.N.A. Plant DS Mini Kit (Omega Bio‐tek) according to the manufacturer's instructions. To ensure genetic homogeneity for future genetic and morphological studies, the same tubers were also used to initiate in vitro cultures of new potato varieties.

The quality of genomic DNA was assessed in a 1% agarose gel polymerized with 1X TBE buffer (89 mM Tris, 89 mM Borate, 2 mM EDTA, pH 8.3). Each sample was prepared by mixing 5 μl of genomic DNA with 1 μl of Blue/Orange loading dye 6X (Promega) and electrophoresed in a 1% agarose gel at 7 V/cm for 1 hr. The gel was stained with 3X GelRed (Biotium) and briefly rinsed with Milli‐Q water (Millipore). The bands were visualized using a Gel Doc XR+ Imaging System (Bio‐Rad) with Image Lab Software (version 6.0). The concentration of genomic DNA for each sample was then determined with a NanoDrop One Spectrophotometer (Thermo Scientific) by measuring the absorbance at 260 nm.

### 2.3 Simple sequence repeat amplification

Simple sequence repeat markers were selected based on the high levels of polymorphism, discrimination ability, and the quality of band scoring from previous studies (Bali et al., 2017; Ghislain et al., 2004, 2009; Karaagac, Yilma, Cuesta‐Marcos, & Vales, 2014). In total, 10 markers were chosen, of which nine were developed by Milbourne et al. (1998) and one was developed by Ghislain et al. (2004). Among them, seven belong to the most recent PGI kit (Ghislain et al., 2009). The sequences for the primers and the SSR characteristics are listed in Table 2.

**Table 2:** SSR markers selected in this study including their repeat motif, chromosome location, the sequences of their specific primers (forward and reverse), and the annealing temperature used in PCR amplification

SSR name	Repeat Motif	Chrom. Location	Primer Sequences (5′ to 3′)	Annealing Temp. (°C)	PGI kit	Ref.
STM0019a,b	(AT)7 (GT)10 (AT)4	VI	F: AATAGGTGTACTGACTCTCAATG	50	Yes	a
	(GT)5 (GC)4 (GT)4		R: TTGAAGTAAAAGTCCTAGTATGTG			
STM0030	Compound	XII	F: AGAGATCGATGTAAAACACGT	58	No	a
	(GT/GC)(GT)8		R: GTGGCATTTTGATGGATT			
STM0031	(AC)5…(AC)3 GCAC	VII	F: CATACGCACGCACGTACAC	55	Yes	a
	(AC)2 (GCAC)2		R: TTCAACCTATCATTTTGTGAGTCG			
STM0037	(TC)5 (AC)6 AA (AC)7 (AT)4	XI	F: AATTTAACTTAGAAGATTAGTCTC	50	Yes	a
			R: ATTTGGTTGGGTATGATA			
STM1016	(TCT)9	VIII	F: TTCTGATTTCATGCATGTTTCC	50	No	a
			R: ATGCTTGCCATGTGATGTGT			
STM1052	(AT)14 GT (AT)4 (GT)6	IX	F: CAATTTCGTTTTTTCATGTGACAC	55	Yes	a
			R: ATGGCGTAATTTGATTTAATACGTAA			
STM1104	(TCT)5	VIII	F: TGATTCTCTTGCCTACTGTAATCG	55	Yes	a
			R: CAAAGTGGTGTGAAGCTGTGA			
STM1106	(ATT)13	X	F: TCCAGCTGATTGGTTAGGTTG	50	Yes	a
			R: ATGCGAATCTACTCGTCATGG			
STM2022	(CAA)3…(CAA)3	II	F: GCGTCAGCGATTTCAGTACTA	50	No	a
			R: TTCAGTCAACTCCTGTTGCG			
STPoAc58	(TA)13	V	F: TTGATGAAAGGAATGCAGCTTGTG	60	Yes	b
			R: ACGTTAAAGAAGTGAGAGTACGAC			

Note

The reference for each SSR marker is indicated with “a” for Milbourne et al. (1998) and “b” for Ghislain et al. (2004).

The polymerase chain reaction was performed in a total volume of 20 μl containing 20 ng of template genomic DNA, 0.5 μM of each primer (forward and reverse), and 1X of a ready‐to‐use solution from HotStarTaq *Plus* Master Mix Kit (Qiagen) containing 1 U of DNA polymerase, buffer, 200 μM of dNTPs, and 1.5 mM of MgCl_2_.

Amplification reactions were performed in a Mastercycler Nexus GX2 thermocycler (Eppendorf) using the following program: an initial step of 5 min at 95°C, followed by 40 cycles of 20 s at 95°C, 20 s at the annealing temperature for each primer set (Table 2), 45 s at 72°C, a final elongation step of 10 min at 72°C, and storage at 4°C. To confirm the amplification reaction, 5 μl of each PCR product was mixed with 1 μl Blue/Orange loading dye 6X and then separated in a 2% agarose gel along with a GelPilot 50 bp ladder (Qiagen). Electrophoresis and detection of PCR products were conducted as explained in the Genomic DNA extraction section.

### 2.4 Denaturing polyacrylamide gel electrophoresis (PAGE)

The amplification products were separated in a 6% denaturing polyacrylamide gel (20 × 45 cm, 0.4 mm thick, with a 20‐well square comb) containing acrylamide:bis‐acrylamide (19:1), 8 M urea, and TBE 1X (Anderson, Wright, & Meksem, 2013). A 2 μl sample of each PCR product was mixed with 2 μl of Gel Loading Buffer II (Denaturing PAGE) (Ambion ‐ Applied System) containing formamide and heated at 95°C for 5 min for denaturation and immediately placed in ice until loaded. The gel was pre‐run at 50 W for 2 hr to reach ~55°C before loading 4 μl of each sample along with a 10 bp DNA ladder (Invitrogen ‐ Life Technology) and a GeneRuler 50 bp DNA ladder (Fisher Scientific). Electrophoresis was then conducted at 55 W for 1.5–3 hr (depending on the expected size of the PCR products) in a vertical Owl S4S Aluminum‐Backed Sequencer System (Thermo Scientific). The gel was stained with 1X GelRed, and the bands were detected as described in the Genomic DNA extraction section.

### 2.5 Data analysis

The size of the PCR products (allele size) was estimated with Image Lab Software (version 6.0) using the 10 bp DNA ladder as a standard. Only strong clear bands were counted to avoid misinterpretation. The SSR alleles were scored by two different persons to increase objectivity. In case of the presence of double or stutter bands, only the size of the upper one was estimated.

The polymorphism information content (PIC), which estimates the allelic diversity, was calculated using the formula *PIC* = 1−∑*p*_*i*_^2^, where *p*_*i*_ is the frequency of *i* ^th^ allele of the SSR locus (Nei, 1973). The power of discrimination (PD) was calculated using the formula *PD* = 1−∑*g*_*j*_^2^, where *g*_*j*_ is the frequency of the *j* ^th^ genotype of the SSR locus (Aranzana, Carbó, & Arús, 2003). The PD gives an estimation of the probability for two individuals to exhibit different allele profiles for the same locus. Jaccard's similarity coefficients (or Jaccard's index) were calculated, recorded in a matrix, and used to design a dendrogram with an online program (DendroUPGMA) using the unweighted pair‐group method with arithmetic average (UPGMA) cluster analysis (http://genomes.urv.cat/UPGMA/;).

## 3 RESULTS AND DISCUSSION

### 3.1 Simple sequence repeats amplification and detection

**Figure 1:**
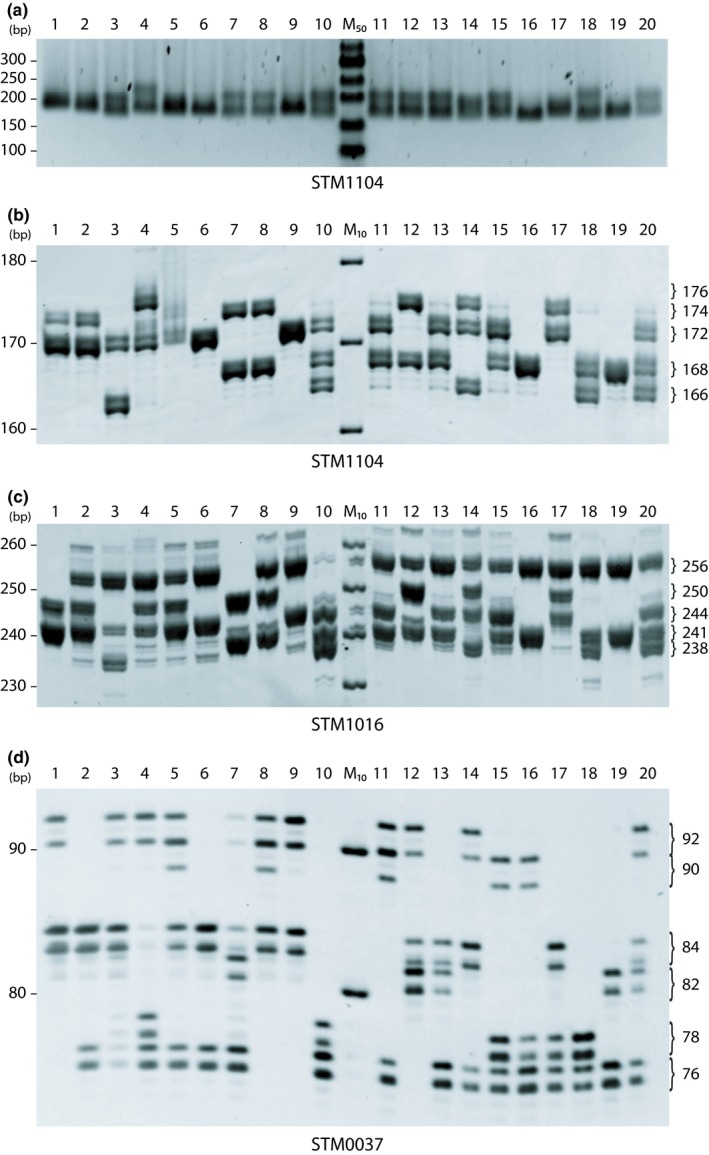
Amplification products of SSR marker STM1104 (a, b), STM1016 (c), and STM0037 (d), generated from DNA of 20 potato genotypes and resolved in a 2% agarose gel (a) and a 6% denaturing polyacrylamide gel (b, c, d). Lanes M_50_ and M_10_: 50 bp and 10 bp DNA ladders, respectively. Lane 1: potato genotype OB3‐A. Lane 2: OB3. Lane 3: Norland. Lane 4: CW2011. Lane 5: Russet Norkotah. Lane 6: Rose Anna. Lane 7: Banana. Lane 8: N1‐WF. Lane 9: Alta Blush. Lane 10: Bintje. Lane 11: Cecile. Lane 12: Cherry Red. Lane 13: Kennebec. Lane 14: Purple Viking. Lane 15: Ranger Russet. Lane 16: Russet Burbank. Lane 17: Russian Blue. Lane 18: Shepody. Lane 19: Spunta. Lane 20: Yukon Gold. Molecular weight DNA markers are shown on the left. Allele sizes are on the right (polyacrylamide gels)

Twenty potato varieties were DNA fingerprinted in this study using 10 SSR markers. Our first step was to evaluate the efficacy of SSR amplification by separating DNA fragments in a 2% agarose gel (Figure 1a). Overall, we obtained good amplification of all potato genomic DNA with all SSR specific primers, and the ranges of the band sizes corresponded to the ranges obtained in previous studies (Ghislain et al., 2004, 2009). Although a difference between some profiles could be detected in a 2% agarose gel, this method was not sufficiently precise to accurately determine the size and the number of the bands. Thus, we discriminated the PCR products in a 6% denaturing polyacrylamide gel, which is a more suitable method because it allows reaching the 1 bp resolution necessary for SSR analysis. The denaturing characteristics of the gel also aid in band size estimation.

### 3.2 Allelic information

Representative gels for STM1104, STM1016, and STM0037 are shown in Figure 1b,c,d, respectively. In this study, the band size varied from 76 bp for STM0037 to 256 bp for STM1016 (Table 3). The allele size differences in a locus were directly proportional to the motif repeat length for nine of the ten SSR markers analyzed. However, the sizes obtained using STM1104 did not follow this pattern. Notably, the repeat motif is a trinucleotide, but the band size differed by a multiple of two nucleotides. Ghislain et al. (2004) previously reported this pattern for the same locus; rather than an error in the size estimation, this phenomenon is likely due to an allele generation caused by a mechanism other than polymerase slippage.

**Table 3:** PCR fragment sizes obtained using the specific primers for the 10 SSR markers with the different potato genomic DNA, and corresponding PIC and PD values

SSR name	Expected product sizes	Observed product sizes	Number of alleles	Number of patterns	Allele sizes	Band score	PIC	PD
STM0019	99–206	186–206	5	7	186, 190, 196, 202, 206	3	0.685	0.815
STM0030	122–168	138–164	6	10	138, 140, 142, 146, 148, 164	3	0.773	0.820
STM0031	185–211	158–192	4	7	158, 174, 192, 194	3	0.674	0.765
STM0037	87–133	76–92	6	14	76, 78, 82, 84, 90, 92	1	0.802	0.915
STM1016	243–275	238–256	5	12	238, 241, 244, 250, 256	1	0.773	0.890
STM1052	214–263	208–224	3	6	208, 216, 224	1	0.643	0.790
STM1104	178–199	166–176	5	10	166, 168, 172, 174, 176	1	0.732	0.875
STM1106	145–211	139–193	5	6	139, 151, 154, 157, 193	2	0.669	0.740
STM2022	173–243	181–196	3	3	181, 187, 196	2	0.520	0.395
STPoAc58	243–263	231–245	2	2	231, 245	3	0.477	0.455

Note

Expected product sizes are taken from Ghislain et al. (2009) and all sizes are expressed in bp. Band score 1: easy to score, 2: medium to score, 3: difficult to score.

All SSR markers in this study exhibited polymorphism. In total, 44 unique alleles were detected for the 10 different loci, and the number of alleles per locus ranged from two for STPoAc58 to six for STM0030 and STM0037, with an average of 4.4 (Table 3). This average number of alleles per locus was similar to those reported by Namugga, Sibiya, Melis, and Barekye (2017) (avg = 3.9), Favoretto, Veasey, and Melo (2011) (avg = 4.6) and Bali et al. (2017) (avg = 4.6), but lower than in Fu et al. (2009) (avg = 6.4), Moisan‐Thiery et al. (2005) (avg = 7.3), and Voronova et al. (2008) (max = 12). In some studies, the number of alleles per locus is even higher, as in the case for STI0023 with 21 alleles (Spooner et al., 2007), which may be explained by the high number of genotypes analyzed (742 potato landraces).

As *Solanum tuberosum* L. is a tetraploid species, one to four alleles per SSR locus were expected for each potato genotype. In total, we obtained 77 unique patterns for all of the SSR markers, including the mono‐, di‐, tri‐, and quadri‐allelic genotypes. Voronova et al. (2008) reported mono‐ and quadri‐allelic genotypes as rare (14% of patterns for both). In our study, quadri‐allelic genotypes were also rare (6.5%), only occurring twice for STM0037 and STM1016, and once for STM0030. However, mono‐allelic genotypes were more common (27.3%) than previously reported (Voronova et al., 2008). Finally, di‐ and tri‐allelic genotypes are also frequent (40.2% and 26%, respectively).

The SSR marker STM0019 has been previously reported to be possibly multilocus because multiple bands were detected with ranges of 83–124 bp (STM0019a) and 155–241 bp (STM0019b) (Ghislain, Andrade, Rodríguez, Hijmans, & Spooner, 2006; Ghislain et al., 2009; Karaagac et al., 2014). In this study, we detected bands only between 186 and 206 bp, which correspond to STM0019b. The absence of bands corresponding to STM0019a may be due to a difference between studies in the annealing temperature during PCR and/or the use of a different DNA fragment detection technique. Ghislain et al. (2006, 2009) used 47°C while we used 50°C. The higher temperature may have prevented the primers from annealing to the second locus. Moreover, IRDye‐labeled M13 forward primer and a LI‐COR DNA Analyzer system, used by Ghislain et al. (2009) and Karaagac et al. (2014), may also allow the detection of more DNA fragments. Although the studies using SSR markers are considered reproducible, the results may vary with different protocols and techniques used.

### 3.3 Evaluation of SSR markers

In this study, the PIC, which is the probability for an individual to be polymorphic for a specific locus, ranged from 0.477 for STPoAc58 to 0.802 for STM0037, with an average of 0.675 per locus (Table 3). Some of the results obtained differed from those of previous studies. For instance, the PIC calculated for STM0037 was 0.802, while this marker has been reported to possess a lower PIC with a value of 0.247 by Namugga et al. (2017) and to be monomorphic by Bali et al. (2017). In this study, the marker STPoAc58 did not exhibit high polymorphism, as we detected only two bands corresponding to our minimum number of alleles per locus and a PIC of 0.477. However, Ghislain et al. (2004) obtained 11 alleles (PIC = 0.754) and Liao and Guo (2014) obtained 14 alleles (PIC = 0.906) for STPoAc58. However, the PIC obtained for the majority of SSR, especially those that gave the best results, were consistent with other studies. We calculated a PIC of 0.773 for STM1016, which was similar to the value of 0.7757 and 0.84 found by Ghislain et al. (2004, 2009). The PIC value for STM1104 ranged from 0.884 to 0.912 in other studies (Ghislain et al., 2004, 2009; Liao & Guo, 2014). Although we obtained a lower PIC for this SSR marker, it had one of the highest PIC calculated in this study at 0.732.

The PD estimates the probability that two randomly chosen individuals show different profiles for the same locus. The PD values ranged from 0.395 for STM2022, to 0.915 for STM0037, with an average of 0.746 per locus (Table 3). The PIC and PD averages (0.675 and 0.746, respectively) indicated that the set of 10 selected SSR markers were informative for differentiating between potato genotypes by fingerprinting. Although PIC and PD do not give the same information, they are closely related to each other and to the number of alleles and patterns. There was a positive correlation between PIC and PD (*r* = 0.94, *p* < 0.001), between PIC and the number of alleles (*r* = 0.92, *p* < 0.001), and between PD and the number of patterns (*r* = 0.87, *p* < 0.001).

We also reported the band scoring according to the ease of band detection and size estimation. Overall, we did not observe clear sharp bands for any of the SSR markers. The main concern was the presence of stutter bands caused by DNA polymerase slippage (Hossienzadeh‐Colagar, Haghighatnia, Amiri, Mohadjerani, & Tafrihi, 2016). A score of 1 was assigned to the SSR markers STM0037, STM1016, STM1052, and STM1104, as we obtained close double bands, which did not interfere with estimating the fragment size (Figure 1b,c,d, for STM1104, STM1016, and STM0037, respectively). A score of 3 was assigned to STM0019, STM0030, STM0031, and STPoAc58 because the presence of a multitude of bands made the band scoring more difficult. Although the band quality obtained can be improved by optimizing PCR conditions, the presence of nonspecific bands remains inevitable.

A set of two markers, containing markers with highest PIC, was the minimum required to discriminate all 20 potato genotypes. Those sets of two markers are STM0019‐STM0037, STM0030‐STM1016, STM0030‐STM1104, STM0037‐STM1016, and STM0037‐STM1104. Based on the PIC and band scoring of each SSR marker, we recommend a set of three loci (STM0037, STM1016, and STM1104) for future genotype analyses of potatoes. The SSR markers STM0037 and STM1104 belong to the PGI kit (Ghislain et al., 2009), which confirms their high capability to distinguish between different genotypes. Although STM1016 is not present the PGI kit, it has been shown to possess a high PIC of 0.840 in another study (Ghislain et al., 2009), and is also recommended by Bali et al. (2017) with a PIC of 0.75. However, more SSR markers could be required to distinguish between two genotypes if they exhibit the same profile using this three loci set.

### 3.4 Differentiation between maternal lines and offspring, and genetic assessment

Only the SSR marker STM0037 was sufficient to distinguish between maternal lines and their respective offspring (i.e., CW2011 and Russet Norkotah, OB3 and Norland, OB3‐A and OB3, and Rose Anna and Banana). The observed different SSR patterns exhibited a clear genetic distinction between the maternal lines and offspring, indicating that the latter may be considered as new cultivars, although they are not yet registered (Table S1).

**Figure 2:**
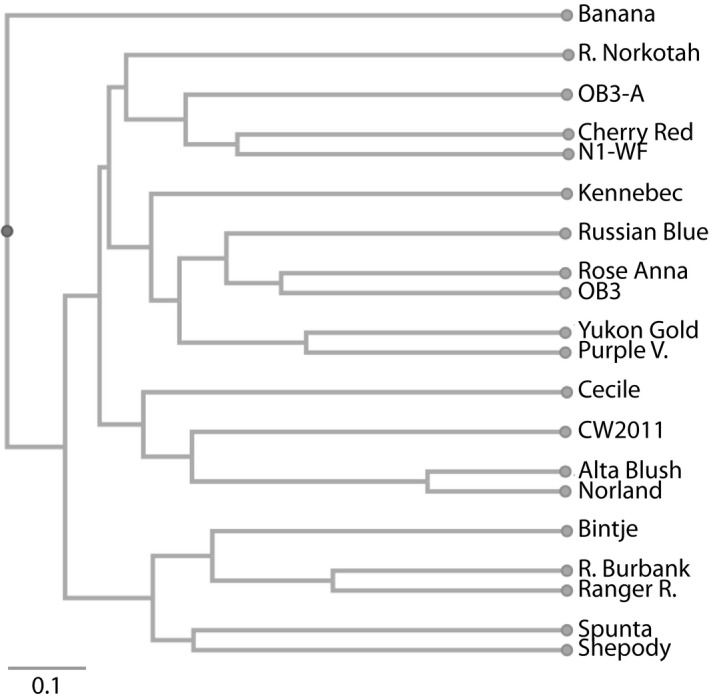
Dendrogram of 20 potato genotypes based on UPGMA cluster analysis and Jaccard's similarity coefficients using allelic diversity data for 10 SSR markers (44 alleles). The generated tree had a cophenetic correlation coefficient of 0.689

Jaccard's similarity coefficients were calculated and used to assess the genetic diversity of the 20 potato genotypes. The coefficients ranged from 0.080 for Alta Blush versus Banana to 0.824 for Alta Blush versus Norland, with an average of 0.397, showing significant genetic diversity among the potato genotypes analyzed. Between maternal lines and offspring, the coefficients were 0.464 for CW2011 versus Russet Norkotah, 0.440 for OB3 versus Norland, 0.609 for OB3‐A versus OB3, and 0.304 for Rose Anna versus Banana. Although there are similarities between the maternal lines and their respective offspring, they do not belong to the same groups based on the dendrogram constructed with the Jaccard's similarity coefficients (Figure 2).

It has been previously shown that progeny of the same maternal line can belong to different clusters on a dendrogram because true seeds of highly heterozygous cultivated potato are genetically different (Liao & Guo, 2014). The opposite is also possible, as two potato genotypes can be genetically highly similar despite different pedigrees (Liao & Guo, 2014). In addition, the construction of a dendrogram depends on the SSR markers selected, and the results obtained from different sets can generate different dendrograms (Liao & Guo, 2014). For these reasons, a tree diagram must be carefully interpreted to determine the lineage of a potato variety. It is difficult to draw definitive conclusions about phylogenetic relationships between the potato genotypes analyzed in this study, because of insufficient information on full parental lineages.

## 4 CONCLUSION

This study evaluated 10 SSR markers on 20 potato varieties and identified the best markers (STM0037, STM1016, and STM1104) regarding their utility in distinguishing between different genotypes. We determined that a set of only two SSR markers was sufficient to differentiate between all 20 potato genotypes. The set of the three best markers can be used for future DNA fingerprinting analyses for breeding programs, cultivar registration, genetic assessment, as well as genotype confirmation to avoid accidental mislabeling and harvest mixture. We also confirmed that SSR markers are simple to use, highly informative, reproducible, and are a good choice for molecular characterization, as found in our case to differentiate between potato genotypes. We used these SSR markers for fingerprinting to confirm the genetic differences between the maternal lines and their offspring generated by open pollination. This information is important to breeders and the future registration of potato genotypes as new cultivars.

## CONFLICT OF INTEREST

The authors declare that they have no conflict of interest.

## AUTHOR CONTRIBUTIONS

A.‐S.T. and D.Y. designed the study and discussed and interpreted the results. A.‐S.T. conducted the experiments and wrote the manuscript. Both authors contributed to the final manuscript.
