# Supplementary material for: Simple sequence repeat analysis of new potato varieties developed in Alberta, Canada
Source: Plant Direct. 2019 Jun 5;3(6):e00140. doi: 10.1002/pld3.140 (PMC6551368; doi:10.1002/pld3.140)
Supplement: Supplementary file 2 [file PLD3-3-e00140-s002.pdf]

University of Lethbridge
Faculty of Arts & Science

Department of Biological Sciences
4401 University Drive
Lethbridge, Alberta, Canada
T1K 3M4
Phone: 403.329.2245
Fax: 403.329.2082

April 23, 2019

Re: Revised Manuscript 2018-00240

Dear Dr. Marna Yandeau-Nelson and
Dr. Ivan Baxter

Thank you very much for your email “Plant Direct: 2018-00240R1: Decision Letter”, dated by April 11, 2019, regarding our manuscript titled “Simple Sequence Repeat Analysis of New Potato Varieties Developed in Alberta, Canada”, by Anne-Sophie Tillault and Dmytro P. Yevtushenko.

You are right, we did not receive the comments provided by Reviewer #2 in your original decision letter. For some reasons, the attachment was not transmitted in the previous correspondence. Therefore, our previous revised manuscript addressed only those comments that were provided by Reviewer #1 and the academic editor.

Here, we have revised the text of manuscript according to the comments of Reviewer #2. We also modified the text of Figure captions to make it more concise and uniform. As before, we highlighted the changes to our manuscript by using yellow text highlighting color. As suggested, we have also included a clean version of the latest version of our manuscript.

We sincerely hope that with these revisions our manuscript will be acceptable for publication in *Plant Direct*.

Our detailed response to the Editorial and reviewers' comments is included below.

Thank you very much for your time and consideration.

We look forward to hearing from you.

Sincerely,

D. Yevtushenko

Dmytro Yevtushenko, Ph.D.
Associate Professor
Department of Biological Sciences
University of Lethbridge, Alberta, Canada
Office: 403-317-2879
Cell: 778-922-3101
Email: dmytro.yevtushenko@uleth.ca

Response to the Reviewer #2 comments:

Reviewer #2: Lines 20-21: Arrange key words alphabetically.

Response. As suggest by the reviewer, we arranged key words alphabetically.

Reviewer #2: Line 43: The FAO, 2006 citation is not listed in the References.

Response. We changed the "FAO 2006" reference for more recent reference of "Devaux et al. 2014" (now Line 48), and listed the new citation in the References.

Reviewer #2: Line 68: "...and evaluating genetic diversity in plants", this would pertain to closely related plants at the species level - not useful for widely divergent species.

Response. We fully agree with the comment. We changed the above phrase for "… and evaluating genetic diversity in plants at the species level" (now Line 76).

Reviewer #2: Lines 73-74: "...possess a specific chromosome location" - this is not always the case, only if mapped.

Response. We fully agree with the comment. We added to the above phrase "(if mapped)" to make our statement more accurate (now Line 82).

Reviewer #2: Line 77: For "...generally conserved within and between related species", this is true only in closely related germplasm, often just at the species level or very closely related species.

Response. As suggest by the reviewer, we modified the above sentence to "Moreover, the sequences flanking an SSR and used to design specific primers are generally conserved in closely related species (Kalia et al. 2011)."

Reviewer #2: Lines 77-80: This sentence states that SSRs are a powerful tool for phylogenetic studies. These are generally not useful for phylogeny of many species.

Response. As suggest by the reviewer, we modified the above phrase to "phylogenetic studies of some species" (now Line 87).

Reviewer #2: Lines 81-83: Regarding "In potatoes, several studies have been conducted to identify and select appropriate SSRs by detecting specific repeat motifs or using enriched genomic libraries and a database of expressed sequence tags", today whole genome sequencing is most commonly used for this purpose.

Response. As suggest by the reviewer, we added the sentence "Nowadays, whole genome sequencing is used for this purpose" (now Lines 92-93).

Reviewer #2: Line 86: Use of the word "developed" would be better than "proposed".

Response. As suggest by the reviewer, we changed the word "proposed" to "developed" (now Line 94).

Reviewer #2: Line 88: Germplasm (no s) would be a better word.

Response. We agree with the comment. We deleted letter "s"-ending from the word "germplasm" (now Line 106).

Reviewer #2: Lines 198-199: Regarding "The polyacrylamide gels were stained with GelRed, and the band sizes were estimated using a 10 bp DNA ladder as a standard", it is not needed to repeat this from Materials and Methods.

Response. We agree with the comment. We deleted that sentence from the Results section of the manuscript.

Reviewer #2: Lines 243-244: The reference is listed here as Liao and Guo, 2013. However, there is only a Liao and Guo, 2014 reference in the References section.

Response. We corrected the above citation to “Liao and Guo (2014)”. It is now Line 272.

Reviewer #2: Line 248: The reference is listed here as Liao and Guo, 2013. However, there is only a Liao and Guo, 2014 reference in the References section.

Response. Similar to the previous comment, we corrected the above citation to “(Liao and Guo, 2014)”. It is now Line 276.

Below is the only comment of Reviewer #2 that we received and replied to in our previous “Response to the Editorial and reviewers' comments:

Reviewer #2: This is an extremely well-written paper with clear English, clearly stated goals, suitable Materials and Methods, and an excellent discussion. Based on the criteria provided to me I recommend acceptance with only minor changes as outlined in comments form on the attached PDF version of the paper.

Response. Thank you very much for your positive comment.

We think that our manuscript has been greatly improved by these revisions and we hope that you will now find it suitable for publication in *Plant Direct*.
